# Receptor Oligomerization and Its Relevance for Signaling by Receptors of the Tumor Necrosis Factor Receptor Superfamily

**DOI:** 10.3389/fcell.2020.615141

**Published:** 2021-02-11

**Authors:** Kirstin Kucka, Harald Wajant

**Affiliations:** Division of Molecular Internal Medicine, Department of Internal Medicine II, University Hospital Würzburg, Würzburg, Germany

**Keywords:** TNF receptor (TNFR) family, TNF ligand superfamily, NFκB, cell death, receptor cluster

## Abstract

With the exception of a few signaling incompetent decoy receptors, the receptors of the tumor necrosis factor receptor superfamily (TNFRSF) are signaling competent and engage in signaling pathways resulting in inflammation, proliferation, differentiation, and cell migration and also in cell death induction. TNFRSF receptors (TNFRs) become activated by ligands of the TNF superfamily (TNFSF). TNFSF ligands (TNFLs) occur as trimeric type II transmembrane proteins but often also as soluble ligand trimers released from the membrane-bound form by proteolysis. The signaling competent TNFRs are efficiently activated by the membrane-bound TNFLs. The latter recruit three TNFR molecules, but there is growing evidence that this is not sufficient to trigger all aspects of TNFR signaling; rather, the formed trimeric TNFL–TNFR complexes have to cluster secondarily in the cell-to-cell contact zone for full TNFR activation. With respect to their response to soluble ligand trimers, the signaling competent TNFRs can be subdivided into two groups. TNFRs of one group, designated as category I TNFRs, are robustly activated by soluble ligand trimers. The receptors of a second group (category II TNFRs), however, failed to become properly activated by soluble ligand trimers despite high affinity binding. The limited responsiveness of category II TNFRs to soluble TNFLs can be overcome by physical linkage of two or more soluble ligand trimers or, alternatively, by anchoring the soluble ligand molecules to the cell surface or extracellular matrix. This suggests that category II TNFRs have a limited ability to promote clustering of trimeric TNFL–TNFR complexes outside the context of cell–cell contacts. In this review, we will focus on three aspects on the relevance of receptor oligomerization for TNFR signaling: (i) the structural factors which promote clustering of free and liganded TNFRs, (ii) the signaling pathway specificity of the receptor oligomerization requirement, and (iii) the consequences for the design and development of TNFR agonists.

## Introduction

The receptors of the tumor necrosis factor (TNF) receptor superfamily (TNFRSF) are of overwhelming importance in the regulation of the immune system but are also involved in the maintenance of tissue homeostasis and development. For example, the two receptors of TNF, TNF receptor-1 (TNFR1) and TNF receptor-2 (TNFR2), regulate the interaction of the various types of immune cells and also the interplay of the latter with practically any type of non-hematopoietic cells; CD40 stimulates antigen-presenting cells; CD27, OX40, 41BB, and RANK costimulate T cells; BCMA, TACI, and BaffR regulate B-cell maturation; CD95 and the two death receptors of TRAIL contribute to tumor surveillance; Fn14 promotes tissue repair; and EDAR drives the development of skin appendages (Aggarwal et al., 2012). The TNFRSF receptors (TNFRs) are characterized by a cysteine-rich domain (CRD) which can be found in their ectodomain in one to six copies (Locksley et al., 2001). The CRDs are involved in ligand binding but can also promote receptor self-assembly. Besides the CRDs, there are no structural features which are present in all TNFRs. However, there are some structural and functional aspects which allow the definition of three functionally and structurally distinct subgroups of the TNFRSF. Most TNFRs contain one or more short binding motifs for proteins of the TNF receptor-associated factor (TRAF) family which link these TRAF-interacting TNFRs to intracellular signaling pathways enabling the activation of transcription factors of the NFκB family and various MAP kinase cascades (Xie, 2013; Park, 2018). A second subgroup of TNFRs, the death receptors, harbors a structurally conserved protein–protein interaction domain in the cytoplasmic part, the so-called death domain (DD) (Siegmund et al., 2017). The DD and the death receptors received their name due to the fact that some DD-containing TNFRs trigger cell death pathways by interaction with cytoplasmic DD-containing proteins. However, despite the name, DD-mediated interactions are also involved in the stimulation of non-cytotoxic signaling pathways by death receptors including TRAF-mediated engagement of NFκBs (Siegmund et al., 2017). Besides the signaling competent TNFRSF subgroups of the TRAF-binding and DD-containing TNFRs, there is a third signaling incompetent subgroup of decoy receptors which comprises soluble receptors, receptors anchored to the plasma membrane *via* a GPI moiety, and a receptor with a non-functional DD.

Besides a very few exceptions, for example p75NGFR, which is stimulated by proNGF, and DR6, which seems to be activated by an N-terminal fragment of the amyloid precursor protein (Lee et al., 2001; Nikolaev et al., 2009), the TNFRs become activated by ligands of the TNF superfamily (TNFSF; Locksley et al., 2001; Bodmer et al., 2002). The TNFSF ligands (TNFLs) form a structurally comparatively homogeneous protein family and are characterized by a C-terminal TNF homology domain (THD), which promotes the assembly into homotrimeric and in a few cases also into heterotrimeric molecules (Bodmer et al., 2002). In the trimeric state, the THD furthermore mediates then the interaction with the receptors of the TNFRSF. Typically, TNFLs are initially expressed as type II transmembrane (TM) proteins, in which the extracellular THD is connected to the TM domain and the intracellular domain by a “stalk” region (Bodmer et al., 2002). Most TNFLs also occur as soluble variants, which emerge from the membrane-bound molecules through proteolytic processing in the “stalk” region. Since the soluble TNFL variants still contain the THD, these molecules are also trimers and are typically still able to interact with high affinity with TNFRs. Noteworthy, the signaling competent TNFRs basically differ in their response to soluble ligand trimers (Table 1). TNFRs of one group, called as category I TNFRs, are robustly activated by soluble ligand trimers. Prominent representatives of the category I TNFRs are TNFR1 and LTβR. TNFRs of a second group, however, failed to comprehensively activate cell death signaling and/or classical NFκB signaling in response to soluble ligand trimers despite high affinity binding (Wajant, 2015). This second group of TNFRs, also named as category II TNFRs, comprises the majority of signaling competent TNFRs and includes many translational interesting TNFRs, such as 4-1BB, CD27, CD40, CD95, Fn14, OX40, TNFR2, and the two TRAIL death receptors (TRAILR1/DR4, TRAILR2/DR5). Intriguingly, some TNFLs interact with TNFRs of both categories. For example, TNF binds with high affinity to TNFR1 and TNFR2, but in contrast to TNFR1, which is efficiently activated by soluble and membrane TNF, TNFR2 becomes only potently stimulated by memTNF (Grell et al., 1995, 1998). Similarly, soluble Baff trimers efficiently interact with the TNFRs BaffR, BCMA, and TACI but only efficiently trigger BaffR signaling (Bossen et al., 2008). Thus, it seems that indeed TNFR-type intrinsic properties, and not the quality of the ligand, determine the responsiveness of TNFRs to TNFLs. Particularly, the inability of category II TNFRs to become fully activated by soluble TNFL trimers cannot be simply caused by the lack of specific sequence information present in the corresponding membrane-bound TNFL variants. This is evident from two fundamental observations/experiences in the field: First, for several category II TNFRs, it has been found that efficient receptor activation takes place when their soluble ligands are presented in plasma membrane-associated form, irrespective of how this is achieved. For example, soluble APRIL, which interacts *via* its THD with the TNFRs TACI and BCMA, contains N-terminally a heparan sulfate proteoglycan binding motif enabling soluble APRIL to bind to proteoglycans (Hendriks et al., 2005; Ingold et al., 2005), such as syndecan-1 (Joo et al., 2012) and syndecan-4 (Jarousse et al., 2011). More important, however, is that proteoglycan-bound APRIL is superior to soluble APRIL in the activation of B cells (Ingold et al., 2005; Kimberley et al., 2009; Joo et al., 2012). Similarly, it has been described that the extracellular matrix protein fibronectin and the keratan sulfate proteoglycan lumican bind soluble CD95L and enhance its ability to trigger apoptosis induction by the death receptor CD95 (Aoki et al., 2001; Vij et al., 2005). Likewise, trimeric soluble TNFL fusion proteins containing an anchor domain, which allows binding to a cell surface-exposed structure, acquire strong category II TNFR-stimulating potency when bound to their anchoring target. The anchoring-dependent mode of receptor activation has been demonstrated for several category II TNFRs (Table 1). Typically, scFv domains recognizing a cell surface-exposed tumor antigen or tumor stroma antigen are used as anchor domain, but the suitability of other types of protein domains has been demonstrated as well [for a review, see, e.g., (de Bruyn et al., 2013; Wajant et al., 2013; Wajant, 2019)]. Worth mentioning and of potential translational importance is the fact that the use of an appropriate anchor domain allows the generation of soluble TNFL fusion proteins which not only ensure full activation of category II TNFRs but also do this in a local fashion and/or link it with a second activity.

**TABLE 1:** Activation of classical NFκB and cell death signaling by category I and category II TNFRs in response to soluble TNF ligands (sTNFLs).

TNFR	Category	TNFL	sTNFL variant/activity (EC_50_ trimer: EC_50_ hexa-, nonamer, etc.)		sTNFL variant/activity (EC_50_ no anchoring: EC_50_ PM anchoring)	
				References		References
BaffR	I	Baff	Flag-Baff/>100	Bossen et al., 2008		
			Baff 64-mer/>100			
DR3	I	TL1A	Flag-TNC-TL1A/1	Bittner et al., 2016		
GITR	I	GITRL	Flag-TNC-GITRL/5	Wyzgol et al., 2009; Richards et al., 2019	Sc40-GITRL/5	Wyzgol et al., 2009
			HERA-GITRL/10			
LTbR	I	LTab_2_	Flag-scLTab_2/_1	Lang et al., 2016		
		LIGHT	Flag-TNC-LIGHT/1	Lang et al., 2016		
TNFR1	I	TNF	Flag-TNF/1	Schneider et al., 1998		
		LTa	Flag-TNC-LTa/1	Lang et al., 2016		
41BB	II	41BBL	Flag-TNC-41BBL/>100	Wyzgol et al., 2009	Sc40-41BBL	Wyzgol et al., 2009
BCMA	II	APRIL	Flag-APRIL/>20	Bossen et al., 2008		
CD27	II	CD27L	Flag-TNC-CD27L/>100	Wyzgol et al., 2009		
CD40	II	CD40L	Flag-CD40L/20	Holler et al., 2003; Wyzgol et al., 2009	Sc40-CD40L/20	Wyzgol et al., 2009; Brunekreeft et al., 2014
			Flag-CD40L/>>100		scFv:EpCAM-CD40L/20	
CD95	II	CD95L	Flag-CD95L/>1,000	Schneider et al., 1998; Holler et al., 2003	Sc40-CD95L/>>100	Samel et al., 2003
			Fc-CD95L/>1,000			
			ACRP-CD95L > 1,000			
EDAR	II	EDA-A1	Flag-EDA-A1/>>100	Swee et al., 2009		
Fn14	II	TWEAK	Flag-TWEAK/>1,000	Roos et al., 2010	Sc40-TWEAK/>>100	Roos et al., 2010
			Fc-TWEAK/>>100			
OX40	II	OX40L	Flag-OX40L/>100	Muller et al., 2008	Sc40-OX40L/>100	Muller et al., 2008
			Fc-OX40L/>20			
TACI	II	APRIL	Flag-APRIL/>100	Bossen et al., 2008		
		Baff	Flag-Baff/>100	Bossen et al., 2008		
			Baff 64-mer/>100			
TNFR2	II	TNF	Flag-TNF/100	Schneider et al., 1998; Prada et al., 2020		
			TNC-scTNF(143N/145R)/>1,000			
TRAILR1	II	TRAIL	Flag-TNC-TRAILmutR1/100	Trebing et al., 2014a	scFv:CD70-TNC-TRAILmutR1/100	Trebing et al., 2014a
TRAILR2	II	TRAIL	Flag-TRAIL/>1,000	Schneider et al., 1998; Wajant, 2019	AD-TRAILs > 100	Wajant, 2019
			Oligomeric TRAILs/>100			

*Please note that this is a non-exhaustive table listing representative reports.*

Second, soluble TNFL molecules convert to potent category II TNFR agonists upon physical linkage of two or more ligand trimers (Table 1). Oligomerization of soluble TNFLs by natural means has for example described for CD95L and Baff. Soluble CD95L present in the bronchoalveolar lavage fluid of patients suffering from acute lung injury turned out unexpectedly to be highly apoptotic (Herrero et al., 2011). It turned out that the soluble CD95L molecules of bronchoalveolar lavage fluid are aggregated due to oxidation. Moreover, the bronchoalveolar lavage fluid of acute lung injury patients promoted oligomerization of recombinant soluble CD95L *in vitro* resulting in an enhanced ability to trigger CD95-mediated cell death (Herrero et al., 2011). Soluble Baff occurs as other soluble TNFLs as a trimeric protein and also in the form of a 60-mer. The Baff 60-mer, however, displays approx. 100-fold higher capacity as trimeric soluble Baff to trigger TACI signaling (Bossen et al., 2008). Oligomerization of soluble TNFL trimers can be straightforwardly achieved with the help of genetically engineered recombinant TNFLs. Introduction of an N-terminal tag, e.g., a Flag tag, allows controlled oligomerization of soluble ligand trimers by treatment with an anti-tag antibody, and fusion with another multimerization domain, besides the THD, often results in the formation of molecules with defined stoichiometry containing, e.g., 6, 9, or 12 TNFL protomers. TNFL fusion proteins, for example, harboring N-terminally the dimerizing Fc domain of human IgG1 typically form hexameric molecules containing two parallel orientated trimeric “TNFL” subdomains (e.g., Holler et al., 2003; Muller et al., 2008; Wyzgol et al., 2009). Over the years, all ligands of the TNFSF have been expressed as soluble Flag-tagged trimers or hexameric Fc-fusion proteins and have been analyzed with respect to their TNFR-stimulating activities by various groups (Table 1). These studies clearly showed that the THD without any other specific sequence information encoded in membrane-bound TNFL molecules is fully sufficient to ensure TNFR binding and TNFR activation, of course in some case only upon oligomerization. Indeed, the absence or demonstration of strongly differing activation of a TNFR by trimeric and aggregated soluble TNFL variants provides the essential experimental evidence for identifying and defining category I and category II TNFRs. It is also worth mentioning that the oligomerization of soluble TNFLs, as far as examined, does not increase their affinity for TNFRs (Fick et al., 2012; Lang et al., 2012). The improved responsiveness of category II TNFRs to aggregated soluble TNFL variants can therefore not simply be attributed to increased receptor occupancy. This is particularly clear from the example of CD95L, since in this case it has even been shown that the soluble ligand variant acts as an inhibitor of its TM counterpart at least in the context of apoptosis induction (Suda et al., 1997).

## TNFR Assembly in the Absence of Ligand

In unstimulated cells, TNFRs are present as monomeric and dimeric or trimeric molecules (Table 2). Dimerization of TNFRs can occur covalently through the formation of cysteine bridges or by non-covalent interactions between specialized parts in the TNFRs not involved in ligand binding. For example, immunoprecipitation experiments with anti-CD27 antibodies revealed a major homodimeric molecule species in T cells (van Lier et al., 1987; Bigler et al., 1988), and immunoprecipitation of p75NTR revealed a mixture of monomeric and cysteine-bridged dimeric receptor species (Vilar et al., 2009). A minor fraction of disulfide-bonded homodimers has also been reported for CD40 in unstimulated B cells (Reyes-Moreno et al., 2004). Noteworthy, CD40 activation enhances covalent CD40 dimerization by promoting the formation of a cysteine bridge *via* C238 located in the cytoplasmic domain of the molecule (Reyes-Moreno et al., 2007). The expression of the 4-1BB ectodomain, furthermore, resulted in a mixture of monomers and C121-linked dimers (Bitra et al., 2018). Noteworthy, it has been furthermore reported that 4-1BB colocalizes with OX40 in activated T cells and also forms immunoprecipitable complexes with this TNFR, presumably again with the help of cysteine bridges (Ma et al., 2005).

**TABLE 2:** Ligand-free assembly of TNFRs.

TNFR	Assembly state	Domain involved	Method	References
CD27	Dimer	Disulfide linked	SDS-PAGE of IPs	van Lier et al., 1987; Bigler et al., 1988
P75NGFR	Dimer	Disulfide linked	SDS-PAGE of IPs	Vilar et al., 2009
CD40	Fraction of dimers	Disulfide linked	Western blot	Reyes-Moreno et al., 2004
41BB	Fraction of dimers	Disulfide linked	SEC	Bitra et al., 2018
CD95	Dimer	AA 1–49 (CRD1)	SEC	Papoff et al., 1999; Siegel et al., 2000
	Dimers + trimers	AA 1–42 (CRD1)	Cross-linking	
			FRET	
TACI	At least trimers		SDS-PAGE of IPs	Garibyan et al., 2007
			Cross-linking	
			FRET	
TNFR1	Trimers	AA 1–54 (CRD1)	Cross-linking	Chan et al., 2000
			FRET	
TNFR2	Trimers	AA 1–54 (CRD1)	Cross-linking	Chan et al., 2000
			FRET	
CD40	Dimer	AA 20–62 (CRD1)	FRET	Chan et al., 2000; Smulski et al., 2013, 2017
			Cross-linking	
TRAILR1			FRET	Chan et al., 2000; Neumann et al., 2012, 2014
TRAILR2		AA 26–41 (CRD1)	Co-IP	Clancy et al., 2005; Neumann et al., 2012, 2014
			FRET	
TRAILR4		AA 27–42 (CRD1)	Co-IP	Clancy et al., 2005; Neumann et al., 2012, 2014
			FRET	
RANK		AA 534–539	Co-IP	Kanazawa and Kudo, 2005
CD30			Co-IP	Horie et al., 2002
Fn14	Minor dimer fraction	Cytoplasmic domain	Cross-linking	Brown et al., 2013

*FRET, fluorescence resonance energy transfer; Co-IP, co-immunoprecipitation; SEC, size exclusion chromatography.*

Most TNFRs, however, seem to auto-associate with non-covalent mechanisms. Most important and best investigated in this context is certainly the preligand binding assembly domain (PLAD). This domain was initially functionally defined in CD95 and roughly comprises the first N-terminal CRD1, which is not involved in CD95L binding but present in several dominant-negative acting CD95 splice variants (Papoff et al., 1996, 1999). Cross-linking experiments, fluorescence resonance energy transfer (FRET) studies, and binding studies with a CD95 deletion mutation only comprising aa 1–49 of the mature receptor, indeed, revealed that the N-terminal part of CD95 promotes self-assembly of the molecule (Papoff et al., 1999; Siegel et al., 2000). In particular, it has been found that heterozygous mutations in CD95 causing the autoimmune lymphoproliferative syndrome (ALPS) interfere with CD95L binding in a dominant-negative fashion, too (Siegel et al., 2000). The dominant-negative effect of CD95L binding-defective mutants and splice variants is difficult to explain if one assumes that CD95L binds to CD95 monomers but becomes straightforwardly understandable if one takes into consideration that CD95L might also bind to pre-assembled dimeric or trimeric receptor species. The dominant-negative effect of CD95L binding-deficient CD95 variants is possibly also of relevance in tumor development as it has been observed that MMP-7 cleaves off a part of the CD95 PLAD resulting in reduced apoptosis sensitivity of tumor cells (Strand et al., 2004). Similarly, it has been demonstrated that the common variable immunodeficiency (CVID)-causing C104R TACI mutant prevents ligand binding but leaves PLAD/CRD1-mediated self-assembly intact (Garibyan et al., 2007). Self-assembly involving the N-terminal CRD1 or parts thereof has also been reported for TNFR1, TNFR2, CD40, TRAILR1, TRAILR2, and TRAILR4 (Chan et al., 2000; Clancy et al., 2005; Smulski et al., 2013; Neumann et al., 2014). Ligand binding-defective TNFR mutants with an intact PLAD may elicit their dominant-negative effect by two mechanisms: first, by decreasing the fraction of dimerized wt TNFR molecules, which often have superior ligand affinity compared with their monomeric counterparts and which therefore might act as the primary ligand binding receptor species; and second, by forming inactive heterocomplexes with liganded cell expressed wt receptor molecules. In view of this mode of action, soluble PLAD-containing protein variants should act as inhibitors of their parental TNFRs. Indeed, dimeric fusion proteins of the PLAD of TNFR1 with glutathione S-transferase or the Fc domain of human IgG1 have been successfully used in preclinical *in vivo* models to treat TNF/TNFR1-driven diseases, such as collagen- and CpG DNA-induced arthritis, skin lesion development in lupus-prone mice, spontaneous autoimmune diabetes, and myelin oligodendrocyte glycoprotein (MOG)-induced encephalomyelitis (Deng et al., 2005, 2010; Wang et al., 2011). However, with a monovalent soluble CRD1/PLAD construct of CD40, a significant agonism has been observed *in vitro* (Smulski et al., 2013). Thus, the quality of the effects of recombinant PLAD constructs could therefore be dependent on the receptor type considered, the valency of the construct, and/or other not yet investigated factors (e.g., receptor density).

An obvious question concerns the strength and specificity of the PLAD–PLAD interaction, but these issues have been only limitedly studied so far. The fact that concentrations in the micromolar range are required for dimerized TNFR1–PLAD constructs to elicit their inhibitory effect on TNF-induced TNFR1 signaling *in vitro* (Deng et al., 2005) suggests that the PLAD–PLAD affinity is rather low. Indeed, cell-free binding assays with immobilized TNFR1 and TNFR2 ectodomains and the monomeric PLAD of TNFR1 revealed half maximal binding of the soluble TNFR1–PLAD to TNFR1 with 9 μM and to TNFR2 with approx. 2 μM (Cao et al., 2011). Likewise, surface plasmon resonance (SPR) analysis revealed a *K*_D_ of 0.6 μM for the binding of the CD40 CRD1/PLAD to the ectodomain of CD40 (Smulski et al., 2013). SPR studies analyzing the interaction between the soluble ectodomains of TRAILR1, TRAILR2, TRAILR3, and TRAILR4, furthermore, revealed affinities between 1 and 10 μM for homotypic and heterotypic interactions (Lee et al., 2005). Low PLAD–PLAD affinities in the micromolar range match well with the fact that soluble TNFR molecules mainly occur as monomers and have thus to be fused with oligomerizing domains, e.g., the Fc domain, to obtain decoy receptors with high apparent affinity (avidity) for their corresponding ligands.

The lack of strong differences in the affinity of the TNFR1–PLAD for TNFR1 and TNFR2 reported in the abovementioned study by Cao et al. (2011) as well as the heterotypic interactions observed for the ectodomains of the various TRAIL receptors suggests that there can be some promiscuity in PLAD–PLAD interactions. Indeed, there is evidence from FRET and co-immunoprecipitation experiments that TRAILR2 and CD95, but not TRAILR1, TACI, BCMA, or BaffR, interact *via* their extracellular domain with CD40 in a competitive manner and so reduce homotypic CD40 dimerization (Smulski et al., 2017). In accordance with these findings, there was attenuated CD40L-induced signaling in cells with increased expression of TRAILR2 and CD95 (Smulski et al., 2017). The lack of discrimination between TNFR1 and TNFR2 in the study with the TNFR1–PLAD is nevertheless quite unexpected. In FRET experiments with intact cells, there was no evidence for an interaction of TNFR1 and TNFR2 (Chan et al., 2000), and in previous co-immunoprecipitation studies, there was no evidence for binding between TNFR1 and TNFR2 as well (Moosmayer et al., 1994; Pinckard et al., 1997). The reasons underlying this contradiction remain to be clarified but could mean that additional factors besides PLAD–PLAD interaction contribute to the specificity of TNFR interactions in the absence of ligand.

**FIGURE 1:**
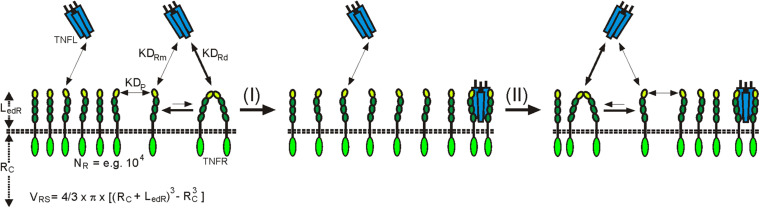
Plasma membrane-associated expression results in high local TNFR concentrations in the TNFR-accessible shell (L_*edR*_) around the cell resulting in dimer formation in the absence of ligand despite low auto-affinity (KD_*P*_). Ligand trimers preferentially bind with higher affinity to dimeric (KD_*RD*_) than to monomeric receptor (KD_*RM*_) species. Liganded TNFR dimers are more stable than ligand-free TNFR dimers and are thus removed from the equilibrium with the monomers. Le Chatelier’s principle promotes then the net formation of new ligand-free receptor dimers. *R*_*C*_, radius of the cell; *V*_*RS*_, volume of the receptor accessible shell.

In view of the weak affinity of PLAD–PLAD interactions, at first glance, the question arises whether a significant fraction of the TNFR molecules of a cell occurs in dimeric or trimeric form to become relevant for ligand binding. There are two factors to consider here: first, the volume which is available to TNFRs inserted into the plasma membrane. This volume is very low, so that high TNFR concentrations can be reached. For example, if one considers an idealized cell with a radius of 10 μm and a plasma membrane surface of 1,560 μm^2^ which expresses 10,000 TNFR molecules with an ectodomain length of 0.1 μm, this results in an effective TNFR concentration of approximately 1.3 μM (Figure 1). Second is the stability of the TNFL–TNFR interaction, which is significantly higher than that of the PLAD–PLAD interaction. The ligand affinity of dimeric TNFRs, and even that of monomeric TNFRs (Lang et al., 2016), is significantly higher than the affinity of the PLAD–PLAD interaction. The ligand-bound TNFR dimers/trimers are therefore withdrawn from the equilibrium between free monomeric and free dimeric or trimeric TNFR species, so that, according to Le Chatelier’s principle, there is net new formation of ligand-free dimeric and trimeric TNFR species, which in turn can be again removed from the equilibrium by ligand binding. Ultimately, over time, this mechanism enables the majority of TNFR molecules to recruit in their dimeric/trimeric form TNFL molecules, even if only a small fraction of the receptors are in the dimeric/trimeric state at a given point in time (Figure 1). In accordance with these considerations, it has been measured by quantitative single-molecule super-resolution microscopy in cells with physiological TNFR1 expression levels that in non-stimulated cells 66% of the TNFR1 molecules are present as monomers and 34% as dimers (Karathanasis et al., 2020). After TNF stimulation, evaluation of the TNF-bound TNFR1 pool revealed in the cited study 13% monomers, a trimeric fraction of 64%, and a significant fraction of TNFR1 molecules even appeared as oligomers (23%). Photoactivated localization microscopy studies with photoactivatable CD95 furthermore showed an incorporation of approx. 50% of the receptor molecules in clusters with two, three, or even more receptors (Fu Q. et al., 2016). However, these values were determined in cells with transient overexpression of CD95 in which the supraphysiological high-expression levels of CD95 lead to unnatural, ligand-independent CD95 activation. It can therefore be assumed that by far fewer CD95 molecules are organized in clusters at physiologically occurring expression levels.

Non-covalent TNFR dimerization/trimerization is not only mediated by the PLAD and might also be promoted by other less well-understood mechanisms. So, it has been described for RANK that self-assembly is dependent on a domain/motif which is located in the TM domain proximal part of its cytoplasmic domain (Kanazawa and Kudo, 2005). Similarly, there is evidence from BS3 cross-linking experiments that Fn14 weakly self-associates *via* its C-terminal tail (Brown et al., 2013). Co-immunoprecipitation experiments also argued for self-association by overexpressed CD30 involving the extra- but also the intracellular domain (Horie et al., 2002). There is furthermore strong evidence that at least some TNFRs can also interact *via* their TM domains. However, this type of interaction seems not to be involved in TNFR assembly in the absence of ligand and instead appears to be important in the context of ligand-induced formation of active TNFR signaling complexes. The corresponding literature will therefore be discussed in the next section.

In sum, although realized by different mechanisms, ligand-independent self-assembly has been demonstrated for most TNFRs. TNFR–TNFR interaction might be of dual relevance for the functioning of TNFRs. On the one side, it can improve the affinity for ligand binding by increasing avidity as discussed above in detail; but on the other side, it might also contribute to the regulation of formation of fully signaling competent TNFL–TNFR clusters as discussed in the following section.

## TNFL-Induced TNFR Complexes

**FIGURE 2:**
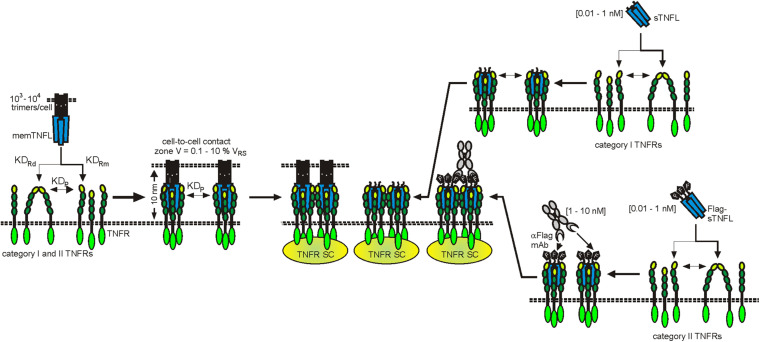
Clustering of TNFRs by membrane-bound and soluble TNFLs. For details, see text.

The X-ray crystal structures of more than 15 TNFL–TNFR complexes have now been published. With the exception of the complex of the heterotrimeric TNFL LTab_2_ with its receptor LTbR, which contains only two receptor molecules, all of these complexes show that a TNFL trimer interacts symmetrically with three receptor molecules (Wajant, 2015). It was therefore initially assumed that a TNFL trimer recruits three receptor molecules and induces the formation of a fully active trimeric receptor signaling complex. The simple finding that some TNFRs (category II TNFRs) bind soluble ligand trimers with high affinity, but, in contrast to membrane-bound TNFSF ligands, do not (or only weakly) stimulate signaling showed that this initial TNFR activation model is in many cases insufficient to reflect experimental reality. The fundamental observation, which was already broadly discussed in the *Introduction*, that category II TNFRs are efficiently activated by soluble TNFLs when they are presented in oligomerized or cell-associated form has led to a two-step model of TNFR activation (Wajant, 2015). According to this model, the secondary interaction of initially formed inactive (or less active) trimeric TNFL–TNFR complexes leads to the formation of oligomeric TNFR clusters, which, unlike the trimeric receptor complexes, are able to effectively activate intracellular signaling pathways (Figure 2). In accordance with this model, it has been observed that membrane-bound TNFL trimers, which are regularly highly active, induce the formation of supramolecular TNFL–TNFR clusters with high efficiency (e.g., Henkler et al., 2005). Factors that may explain the superior cluster-inducing potency of membrane-bound TNFLs are the reduced mobility of the membrane-associated ligands, the alignment of the ligand molecules caused by their membrane-associated state, and certainly also their high “local” concentration in the cell–cell contact. For example, when all TNFR molecules of a spherical cell with a radius of 10 μm, which expresses 10,000 receptors, are bound by the ligands of a neighboring memTNFL expressing cell in a 0.01-μm distance cell–cell contact, which comprises 0.1–10% of the cell surface, a local TNFR concentration of 10–1,000 μM is reached (Figure 2). At these high concentrations, even low TNFR auto-affinities, e.g., due to PLAD–PLAD interactions, are sufficient to ensure secondary clustering and, thus, receptor activation.

## Assembly of Liganded TNFRS

Nuclear magnetic resonance spectroscopy and biochemical studies with the TM domain of CD95 reconstituted in lipid bicells revealed the formation of stable trimers, and CD95 variants harboring mutations disrupting trimerization of the TM domain showed reduced apoptosis induction (Fu Q. et al., 2016). It is worth mentioning, however, that PLAD-mediated self-assembly of CD95 remained intact in these CD95 mutants (Fu Q. et al., 2016). This suggests that the TM domain-driven trimerization of CD95 is not crucial for the assembly of ligand-free receptors and only contributes to the formation of an active CD95L–CD95 signaling complex after ligand binding by not yet clarified mechanisms. The NMR structure of the TM domain of the CD95-related death receptor TRAILR2/DR5 reconstituted in lipid bicells showed surprisingly poor similarity to that of CD95. Admittedly, the TRAILR2 TM domain migrates in SDS-PAGE analysis like the CD95 TM domain as a trimer; in the lipid bicells, however, the TRAILR2 TM domain is packed as a hexamer which is formed by the interplay of a trimerizing and a dimerizing interface present in the TM domain (Pan et al., 2019). TRAILR2 TM domain mutants with a defective dimerization interface still form trimers which are similar in structure to the CD95 TM domain trimers (Pan et al., 2019). It is tempting to speculate, and in accordance with the structural data, that in the plasma membrane, without the space restraints given by the lipid bicells, the TRAILR2 TM domain forms a dimer–trimer network (Pan et al., 2019). TRAILR2 variants harboring mutations destroying either the dimerization or the trimerization interface of the TM domain interfere with apoptosis induction and clustering of overexpressed receptor molecules but not with ligand-independent self-assembly (Pan et al., 2019). Most intriguingly, a genetically engineered TRAILR2 variant with a tobacco etch virus (TEV) protease cleaving side between the TM domain and the TRAILR2 ectodomain induces apoptosis in the absence of ligand upon cleavage with the TEV protease (Pan et al., 2019). This suggests that the unliganded TRAILR2 ectodomain prevents TM domain-driven clustering and activation of TRAILR2. Similar initial observations have been made with TNFR2 and OX40 variants with a TEV protease cleavable ectodomain (Pan et al., 2019).

## Apoptosis Induction and Activation of the Classical NFκB Pathway by Secondary Clustering of Liganded Category II TNFR Trimers

**FIGURE 3:**
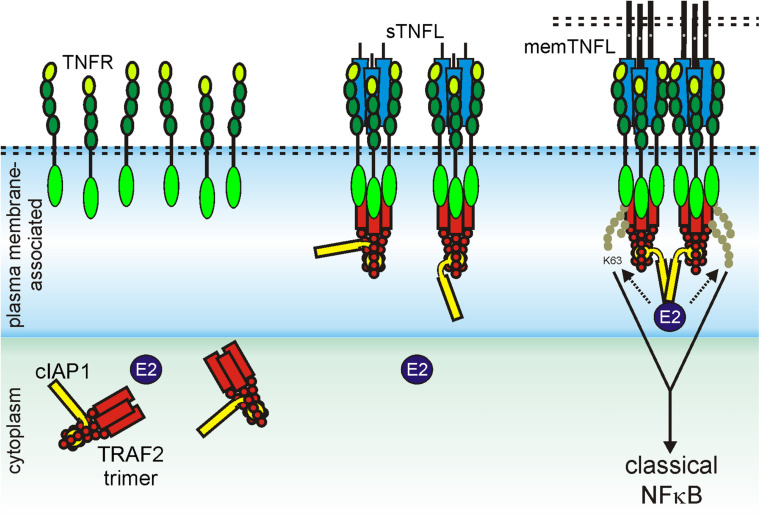
Scheme of TRAF2-interacting TNFR-induced activation of the classical NFκB pathway. For details, see text.

The necessity of secondary aggregation of trimeric TNFL–TNFR complexes for the activation of the classic NFκB signaling pathway and apoptosis induction can be straightforwardly explained from the current knowledge about the molecular mechanisms on how TRAF and DD adapter proteins act in these pathways. The TRAF2 adapter protein occurs as a homotrimeric molecule or as a heterotrimeric molecule in complex with TRAF1 (Xie, 2013). The TRAF1 and TRAF2 protomers share a C-terminal TRAF domain which comprises a coil–coil N-TRAF subdomain mediating trimerization followed by a C-terminal C-TRAF subdomain which contains a TNFR binding site (Xie, 2013; Park, 2018). Homotrimeric TRAF2 and TRAF1–TRAF2 heterotrimers interact with two of their three protomers (2xTRAF2 or TRAF1–TRAF2) in an asymmetric fashion with the baculoviral IAP repeat (BIR) 1 domain of a single monomer of the E3 ligase cIAP1 or the E3 ligase cIAP2 (Mace et al., 2010; Zheng et al., 2010). Monomeric cIAPs exist in an autoinhibited state that prevents the RING domain of the molecule from promoting dimerization. The activation of the E3 ligase activity of the cIAPs is based on the dimerization of the RING domain enabling the interaction with E2 proteins and subsequent K63 ubiquitination of signaling proteins involved in the stimulation of the classic NFκB signaling pathway through TNFRs (Dueber et al., 2011; Feltham et al., 2011; Varfolomeev et al., 2012). Most TRAF-binding TNFRs have one binding site for a protomer of TRAF1, TRAF2, TRAF3, or TRAF5; some TNFRs have in addition a TRAF6 binding site (Table 3). The three receptor molecules of a trimeric TNFL–TNFR complex thus interact with the C-TRAF domain of three protomers of a single TRAF2 homotrimer or a TRAF1/TRAF2 heterotrimer. Accordingly, a trimeric TNFL–TNFR complex only recruits a single and, therefore, inactive, cIAP1 (or cIAP2) molecule, which is not sufficient to efficiently stimulate the classical NFκB signaling pathway (Figure 3). In clusters of two or more trimeric TNFL–TNFR complexes, however, active cIAP1 or cIAP2 dimers can be formed due the close neighborhood of receptor-bound 3:1 TRAF–cIAP complexes so that the classical NFκB signaling pathway can be strongly activated (Figure 3).

**TABLE 3:** TRAF-binding sites in TRAF-interacting TNFRs.

TNFR	Method	TRAF1/2/3/5 site	References
41BB	Two hybrid system (THS)	E236–E249	Arch and Thompson, 1998
BaffR	Receptor mutants IP	P117–D122 + AA 160–183	Xu and Shu, 2002; Ni et al., 2004
	Crystal structure		
BCMA	Receptor mutants IP	A 119–143	Hatzoglou et al., 2000; Granja et al., 2017
	Homology	A165–E168	
CD27	Receptor mutants IP	R238-250	Yamamoto et al., 1998
CD30	GST-receptor mutants IP	V575–G583 + P558–T565	Boucher et al., 1997; Lee et al., 1999
CD40	GST-receptor mutants IP	P230–V241	Lee et al., 1996; Lu et al., 2003
	THS		
GITR	THS	E202–E213	Esparza and Arch, 2005
Fn14	THS	P113–E116	Brown et al., 2003
LTbR	Receptor mutants IP	P389–H402	Force et al., 2000
OX40	THS	T256–E261	Arch and Thompson, 1998
RANK	GST-receptor mutants IP	P607–Q611	Galibert et al., 1998; Kim et al., 1999
TACI	Homology	P270–E273	Granja et al., 2017
TNFR2	Crystal structure	Q420–E427	Park et al., 1999
TROY	Homology	T276–E279	Kojima et al., 2000
XEDAR	Receptor mutants IP	AA 249–254 + AA 273–281	Sinha et al., 2002

**FIGURE 4:**
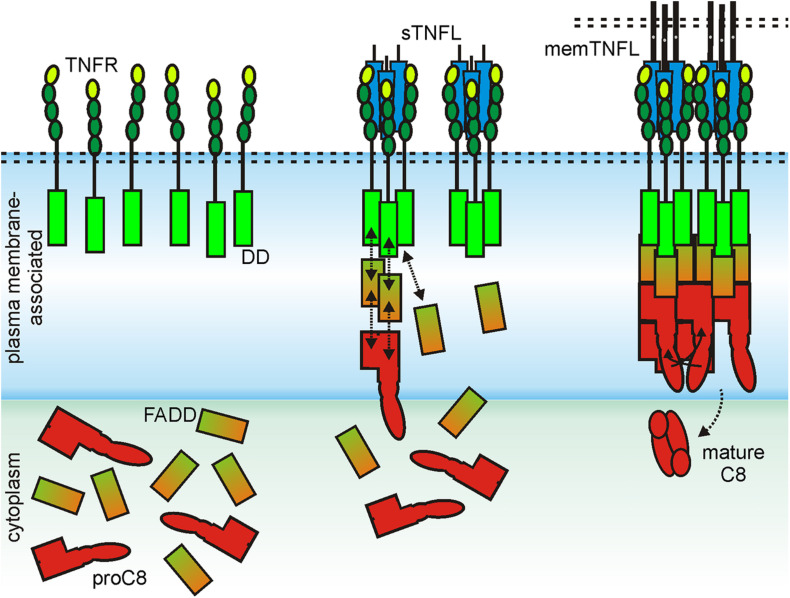
Scheme of CD95-induced caspase-8 activation. For details, see text.

For the initiation of the extrinsic apoptotic signaling pathway through some receptors of the death receptor subgroup of the TNFRSF, the dimerization of an inactive monomer is also necessary, namely that of the procaspase-8 molecule. In this case, too, structural data that were obtained for the DD of the death receptor CD95, the adapter molecule Fas associated death domain protein (FADD), the prodomain of caspase-8, and the complexes of these molecules suggest that at least two trimeric ligand–receptor complexes must come together in order to dimerize procaspase-8 to trigger activation of this enzyme and to engage the apoptotic signaling cascade (Carrington et al., 2006; Scott et al., 2009; Wang et al., 2010; Shen et al., 2015; Park, 2019). Indeed, it has been found that the prodomain of caspase-8 forms filaments which consist of three parallel helical prodomain strands (Fu T.M. et al., 2016). Furthermore, it has been observed that complexes of the CD95 DD and the adapter protein FADD serve as condensation nuclei for the formation of these filaments (Fu T.M. et al., 2016). Now, the adapter protein FADD, which consists of a DD and a death effector domain (DED), interacts with its DD with the DD of CD95 and with its DED with the caspase-8 prodomain. The latter, however, consists of two DEDs that interact in an asymmetrical manner with the single DD of two FADD molecules. Thus, to form the cap of a caspase-8 prodomain filament, six FADD molecules and therefore consequently six CD95-DDs are necessary (Fu T.M. et al., 2016). The formation of a CD95-FADD cap, which stimulates the assembly of procaspase-8 filaments, in which dimerization of two caspase-8 molecules can occur, can therefore explain the need of CD95 clustering required for robust CD95-induced apoptosis (Figure 4). The importance of the secondary interaction of two or more trimeric TNFL–TNFR for the efficient stimulation of the classical NFκB signaling pathway and extrinsic apoptosis obviously does not reflect any fundamental intrinsic receptor limitation. Rather, it is the special signaling pathway-specific way how the signaling proteins involved stimulate inactive enzymes that makes receptor clustering so important in these two examples. TNFRs of category II are not or hardly able to induce the classical NFκB signaling pathway or apoptosis after stimulation with physiological concentrations of soluble ligand trimers. Category I TNFRs, however, such as TNFR1, DR3, GITR, and LTβR, activate these signaling pathways maximally already at low concentrations of soluble ligand trimers. Moreover, further cross-linking of the soluble ligand molecules fails to further enhance their activity (Bittner et al., 2016; Lang et al., 2016). The obvious question for category I TNFRs is, therefore, why in the case of this receptor type the mere binding of soluble ligand trimers is sufficient to achieve maximum and extensive receptor activation. At least in the case of TNFR1 and BaffR, there is evidence that this is due to an increased intrinsic ability of the receptor molecules to self-aggregate. Studies evaluating the functional properties of chimeric receptors composed of the extracellular domain and TM domain of the category I TNFR TNFR1 and the intracellular domain of the category II TNFR CD95 showed strong recruitment of FADD and caspase-8 and apoptosis induction by soluble TNF (Krippner-Heidenreich et al., 2002). However, a chimeric receptor composed of the extracellular and TM domain of the category II TNFR2 and the cytoplasmic CD95 domain needed cross-linking of sTNF for robust signaling (Krippner-Heidenreich et al., 2002). Similarly, chimeric receptors composed of the extracellular domain of the category I TNFR BaffR and the cytoplasmic domains of the category II TNFRs CD95 or TRAILR2 triggered efficient cell death in response to soluble Baff trimers in Jurkat and rhabodmyosarcoma cells (Schuepbach-Mallepell et al., 2015). Thus, transfer of the extracellular and the TM domain of a category I receptor was fully sufficient in this example to overcome the requirement for soluble ligand oligomerization to trigger category II TNFR signaling. Follow-up experiments with the TNFR1-CD95 and TNFR2-CD95 chimeras gave furthermore evidence that the stalk region separating the CRDs from the TM along with the TM crucially contributes to the need of category II TNFRs for cross-linking of soluble ligand trimers to become activated. Transfer of the stalk–TM region of TNFR2 to TNFR1-CD95 was sufficient to reconstitute the need for soluble ligand oligomerization to trigger CD95 signaling (Richter et al., 2012). Vice versa, insertion of the stalk–TM region of TNFR1 into TNFR2-CD95 was sufficient to convert this category II TNFR chimera into a category I receptor (Richter et al., 2012). TM replacement experiments with TNFR1-CD95 and the TMs of TRAILR1 and TRAILR2 furthermore suggest that the TM might affect clustering efficacy, too (Neumann et al., 2012). The simplest explanation of this observation is, of course, that the extracellular and TM domain of category I TNFR, such as TNFR1 and BaffR, has its own considerable intrinsic clustering ability. In view of the evidence discussed above that the extracellular region of category II TNFRs TNFR2 and TRAILR2 antagonizes clustering of liganded receptor trimers (Krippner-Heidenreich et al., 2002; Schuepbach-Mallepell et al., 2015), it is tempting to speculate that this TNFR type does not simply lack clustering ability but rather has evolved repulsive mechanisms to prevent PLAD-driven clustering of soluble ligand-bound TNFRs.

## Signaling Pathway-Specific Oligomerization Requirements of Category II TNFRS

As already discussed above, the fact that two or more trimeric TNFL–TNFR complexes have to aggregate in order to ensure robust activation of the classical NFκB signaling pathway or the extrinsic apoptotic signaling pathway is straightforwardly explained by the signaling pathway-specific requirements for the activation of enzymes (cIAPs, caspase-8), which are indirectly recruited to the TNFRs. The aggregation of liganded TNFRs therefore does not necessarily reflect a factor that is a general prerequisite for the activation of any TNFR-engaged intracellular signaling pathway. In fact, for the category II TNFRs Fn14 and CD95, activities have been described which are already maximally stimulated by soluble ligand trimers.

**FIGURE 5:**
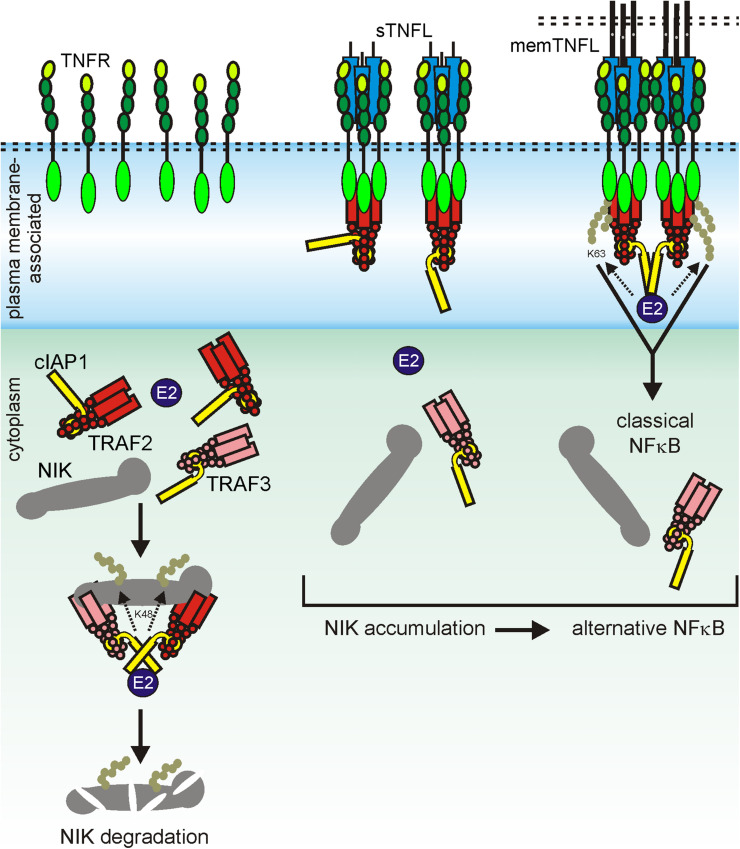
Scheme of sTWEAK-induced activation of the alternative NFκB pathway. In non-stimulated cells, cytosolic TRAF2 and the cIAPs are engaged in NIK degradation, thus in suppression of the alternative NFκB pathway (left panel). Recruitment of TRAF2 and cIAPs to Fn14, irrespective whether triggered by soluble TWEAK or membrane TWEAK, results in reduced cytosolic TRAF2 and cIAP levels and thus reduced suppression of NIK degradation resulting in the activation of the alternative NFκB pathway by accumulated constitutively active NIK.

A systematic and comprehensive analysis with soluble TWEAK (sTWEAK) trimers; oligomeric and hexameric sTWEAK variants; an scFv-sTWEAK fusion protein, which is able to bind to a plasma membrane-presented antigen; and memTWEAK revealed that all sTWEAK variants trigger activation of the alternative NFκB pathway (NIK accumulation, p100 to p52 processing) with similar dose dependencies and reach comparable pathway activity as upon stimulation with memTWEAK-expressing cells. Thus, neither physical connection of two or more sTWEAK trimers nor their anchoring to the plasma membrane resulted in a further enhancement of the ability of sTWEAK to stimulate this Fn14 response (Roos et al., 2010). In contrast, the various TWEAK variants split into two groups with respect to their ability to stimulate the classical NFκB pathway. Hexameric Fc-sTWEAK, oligomerized sTWEAK, and cell surface-anchored scFv-sTWEAK activated the classical NFκB pathway as efficiently as memTWEAK, while sTWEAK and free scFv-sTWEAK showed only at high concentrations a modest stimulatory effect (Roos et al., 2010). It turned out furthermore that irrespective of their oligomerization state and cell surface anchoring, all sTWEAK variants and memTWEAK induce the disappearance of TRAF2 from the cytoplasmic soluble compartment which explains the shared ability to activate the alternative NFκB pathway as follows. As already discussed above, a TRAF2 trimer associates with a single cellular inhibitor apoptosis 1 (cIAP1) or cIAP2 molecule. In the cytoplasm of unstimulated cells, the TRAF2–cIAP1/2 complexes interact with a complex of TRAF3 and the kinase NIK (Xie, 2013; Sun, 2017). The latter activates IKK1 which in turn triggers processing of the NFκB precursor protein p100 to p52 resulting in the nuclear translocation of p52-containing transcription factors and transcription of target genes of the alternative NFκB pathway (Xie, 2013; Sun, 2017). In the TRAF–cIAP–NIK complex, the cIAPs K48-ubiquitinate NIK trigger thereby the proteasomal degradation of NIK resulting eventually in the constitutive active suppression of the alternative NFκB pathway. The sole recruitment of a TRAF2 trimer and its single associated cIAP molecule to sTWEAK-liganded Fn14 without cIAP transactivation is thus already fully sufficient to interrupt the constitutively ongoing inhibition of the alternative NFκB pathway (Figure 5). It is obvious that clustering of the liganded TRAF2–cIAP-containing Fn14 complexes does not result in a further reduction of the cytoplasmic available pool of TRAF2–cIAP1 and TRAF2–cIAP2 complexes and, thus, does not enhance alternative NFκB signaling.

In accordance with the well-established finding that soluble CD95L binds CD95 but does not trigger CD95 clustering and apoptosis, it has been described that sCD95L acts as an inhibitor of memCD95L-induced apoptosis (Suda et al., 1997). However, sCD95L can stimulate Ca^2+^ signaling and migration of myeloid cells, T cells, and various tumor cells (Siegmund et al., 2017). There is evidence that this occurs by DD-dependent and DD-independent pathways which, in contrast to apoptosis induction, do not need FADD and caspase-8 (Tauzin et al., 2011; Poissonnier et al., 2016). The DD-independent mode of Ca^2+^ signaling and the stimulation of cell motility have been traced back to recruitment of PLCγ1 and the tyrosine kinase Yes to a calcium-inducing domain preceding the DD of CD95. The composition and stoichiometry of the sCD95L-induced cell migration-inducing CD95 signaling complex is quite different from the memCD95L-induced apoptotic signaling complex. In the case of TWEAK, it is obvious that the membrane-bound ligand also triggers the signaling events engaged by the soluble ligand. In the case of CD95L, this issue has not been clarified yet. Thus, it is unclear whether memCD95L simultaneously triggers the recruitment of the cell motility-inducing molecules along with FADD and caspase-8 or whether these signaling molecules are utilized by CD95 in an exclusive manner.

## Cell Intrinsic Factors Control TNFR Clustering and Activation

Tumor necrosis factor receptor preassembly, ligand-induced receptor trimerization, and clustering of liganded receptor trimers occur in the complex environment of cellular membranes. It is therefore presumably not surprising that various cellular factors have been identified which regulate TNFR activation by direct or indirect modulation of the clustering process. For example, especially for the TRAIL death receptors and CD95, there is broad evidence that O- and N-glycosylation affect their death-inducing activity. O-glycosylation enhanced ligand-induced clustering of TRAILR1 and TRAILR2 (Wagner et al., 2007). Cancer cells frequently express membrane proteins with truncated *O*-glycans. In the case of the TRAIL death receptors, this results in reduced receptor clustering and, thus, in reduced sensitivity for apoptosis induction (Zhang B. et al., 2019; Jiang et al., 2020). For TRAILR1, it has been further shown that *N*-glycosylation promotes ligand-induced clustering, too (Dufour et al., 2017). Thus, glycosylation *per se* seems to act as a factor which contributes to the constitution of a “normal” interaction competence of TRAIL death receptors enabling efficient ligand-stimulated receptor clustering and formation of cell death-inducing receptor complexes. Noteworthy, glycosylation makes TRAIL death receptors also accessible for carbohydrate-binding proteins. Indeed, there is evidence that galectin-3 traps TRAIL death receptors in glycan nanoclusters and prevents the TRAIL-induced formation of apoptotic receptor complexes (Mazurek et al., 2012). CD95 is also N- and O-glycosylated (Seyrek et al., 2019). In the case of this death receptor, however, it has been reported that inhibition of glycosylation showed only a minor effect on receptor clustering and cell death induction (Shatnyeva et al., 2011) or that it even enhanced cell death induction (Charlier et al., 2010). In the latter study, whether this was again due to the interaction with galectin-3 or another carbohydrate-binding protein remained, however, unclear. There are also reports giving evidence that galectins also interact with the category II TNFRs CD40 and 41BB and the category I TNFR DR3 (Vaitaitis and Wagner, 2012; Madireddi et al., 2014, 2017). Galectin-9 has been found to interact with the CRD4 of 41BB in a carbohydrate-dependent manner without interfering with 41BBL binding. More importantly, the lack of galectin-9 resulted in reduced 41BB-mediated costimulation of CD8^+^ T cells (Madireddi et al., 2014). Likewise, interaction of galectin-9 with DR3 has been demonstrated and correlated with reduced DR3-induced production of IL2 and IFNg in T cells in galectin-9 KO T cells (Madireddi et al., 2017). The functional consequences of the galectin-9–CD40 interaction have only been limitedly studied, but in this case, the interaction correlated with reduced CD40-dependent activity (Vaitaitis and Wagner, 2012). Another type of modification, which could be implicated in the clustering of TNFRs, is palmitoylation. Intracellular palmitoylation near the TM domain of CD95 in L12.10.mFas cells has been reported to promote constitutive lipid raft association of CD95 and CD95L-induced association of CD95 with actin cytoskeleton-linked lipid rafts leading to the assembly of the caspase-8-activating CD95 receptor signaling complex (Feig et al., 2007). It has been, however, not clarified yet how CD95 palmitoylation affects ligand binding and clustering of liganded CD95 complexes in detail. Investigation of this issue is also challenging in view of the observation that CD95 palmitoylation prevents lysosomal degradation of CD95 resulting in higher CD95 expression levels (Rossin et al., 2015). Palmitoylation has also been reported for the TNFRs TRAILR1, TNFR1, the low-affinity NGFR, and DR6 (Vesa et al., 2000; Klima et al., 2009; Rossin et al., 2009; Zingler et al., 2019). In the case of TRAILR1, palmitoylation has again been implicated in lipid raft association, whereas there was no evidence for such an effect in the case of DR6 (Klima et al., 2009; Rossin et al., 2015). The relevance of palmitoylation of TNFR1 and the low-affinity NGFR for ligand binding and receptor clustering has not been investigated yet (Vesa et al., 2000; Zingler et al., 2019). The effects of palmitoylation on the clustering and activation of TNFRs appear mainly to be mediated by controlling the association with lipid rafts. Indeed, the latter has been implicated in manifold studies in the activation of certain TNFRs but often with cell type-specific and/or agonist type-specific relevance. For this special aspect, one is therefore referred to corresponding reviews (e.g., Muppidi et al., 2004; Gajate and Mollinedo, 2015). In sum, although the relevance of receptor modifications and the “plasma membrane environment” for clustering and activation of TNFRs has been demonstrated in many studies for selected TNFRs, the importance for most receptors of the TNFR family has not been addressed so far and many aspects are still unclear. Indeed, even in the broadly investigated cases of CD95 and the TRAIL death receptors, it is largely unknown whether and if yes to which extent the effects of these factors are cell type-, pathway-, or agonist-specific.

## TNF Receptor Activation Requirements: Consequences for the Design and Development of TNFR Agonists

Due to the relevance of TNFRs in immune regulation and maintenance of tissue homeostasis, both the inhibition of TNFRs and the activation of TNFRs can have beneficial therapeutic effects (Aggarwal et al., 2012). The inhibition of TNFRs is comparatively easy to achieve with the help of neutralizing anti-TNFL antibodies or by using decoy receptors, which contain the extracellular ligand binding domain of TNFRs. In fact, several such reagents have been approved for clinical use in various autoimmune diseases and, in particular, include various TNF blockers. In contrast, the therapeutic success of TNFR-activating reagents is so far rather modest. Although TNFR activation appears very attractive for cancer therapy and has indeed been evaluated in this respect in a plethora of preclinical and clinical trials since more than two decades, only recombinant TNF (Beromun) has been approved for clinical use, and this is only for the treatment of soft tissue sarcoma in isolated limb perfusion, a rather rare application. Noteworthy, a not yet approved Fc fusion protein of EDA1 has been successfully used for *in utero* therapy of X-linked hypohidrotic ectodermal dysplasia and restored sweating ability (Schneider et al., 2018). The disappointing clinical success of therapeutic reagents, particularly antibodies, acting by TNFR stimulation is at least partly related to the difficulties in the development of potent TNFR agonists which result from the special molecular mechanisms of TNFR activation described above.

Despite the approval of recombinant soluble TNF for the treatment of soft tissue sarcoma, the potential clinical use of recombinant soluble TNFLs is limited in several ways: First, due to their small size, soluble TNFLs are rapidly cleared from the circulation. For example, for soluble TNF, serum half-life of 6–7 min has been found in mice, and for soluble TRAIL, a serum half-life of 23–31 min has been reported in non-human primates (Beutler et al., 1985; Kelley et al., 2001). Second, category II TNFRs are not or only poorly activated by binding of soluble ligand trimers (see above). These two limitations can be overcome by genetic fusion of soluble TNFLs with heterologous protein domains improving serum retention and/or connecting two or more trimers or enabling cell surface anchoring. The development of soluble TNFL variants with good serum retention and high TNFR agonism was mainly advanced for TRAIL and immunostimulatory TNFLs, such as CD40L, etc. Accordingly, a large number of different TNFL fusion protein formats with considerable agonistic activity and often also good serum retention have been described to date. The various TNFL formats including their mode of action have been comprehensively reviewed recently (e.g., for TRAIL, see, de Bruyn et al., 2013; Wajant, 2019) and will therefore not be discussed here in detail. Several of these highly active soluble TNFL variants have been successfully evaluated in preclinical models for cancer treatment. However, TNFL fusion proteins are typically less efficiently produced as antibodies and often elicit antibody responses, and in general, there is less experience with the translational development and approval of such reagents. Agonistic antibodies are therefore still the means of choice when therapeutic TNFR activation is considered.

**FIGURE 6:**
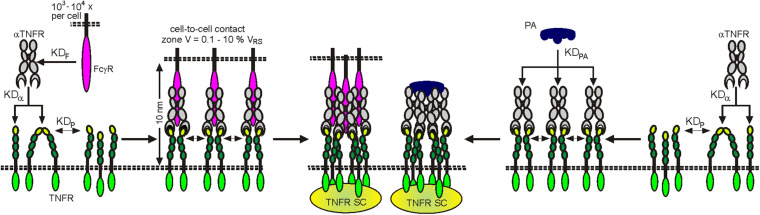
Clustering and activation of TNFRs by antibodies (aTNFRs). Antibodies bind with medium to high affinity to FcγRs (KD_*F*_) and also with affinity to TNFRs (KD_*a*_). The FcγR-bound aTNFRs act then like memTNFLs. Protein A binds multiple aTNFR molecules with high affinity (KD_*PA*_). The resulting complexes act then as oligomeric sTNFLs.

Already in the early 2000s, studies with FcγRIIb-deficient animal models showed that the *in vivo* agonism of CD95 antibodies is dependent on FcγR binding (Jodo et al., 2003; Xu et al., 2003). These observations were perceived as anecdotal reports and initially did not result in consideration in the development and *in vivo* functional analysis of anti-TNFRs. In the last decade, however, a growing list of studies exploiting FcγR-deficient animals and/or antibody variants with defective FcγR binding gives clear evidence for the idea that FcγR-dependent agonism is rather the rule than the exception for antibodies targeting 4-1BB, CD27, CD40, CD95, Fn14, OX40, TNFR2, TRAILR1, and TRAILR2 (Li and Ravetch, 2011, 2012, 2013; White et al., 2011, 2014; Wilson et al., 2011; Salzmann et al., 2013; Trebing et al., 2014b; Dahan et al., 2016; Medler et al., 2019; Zhang P. et al., 2019). To get a first impression to what extent FcγR-dependent agonism is a general phenomenon in the TNFRSF, we evaluated a panel of approx. 30 antibodies, targeting 11 different types of TNFRs, for their FcγR-dependent activity using the same methodology. This study came up with a clear and obvious correlation. Eight of eight antibodies specific for category I TNFRs LTβR and TNFR1 elicit robust agonistic activity irrespective of FcγRIIB binding (Medler et al., 2019; patent WO2019129644). In contrast, all antibodies targeting category II TNFRs—4-1BB, CD27, CD40, CD95, Fn14, OX40, TNFR2, TRAILR1, and TRAILR2—turned out to be largely inactive but converted to strong agonist provided there was the possibility to bind to FcγRIIB (Medler et al., 2019; patent WO2019129644). Only one antibody which targeted GITR showed no agonism at all despite FcγR binding. Noteworthy, the maximum receptor activation reached with the FcγRIIB-anchored anti-TNFR antibodies was comparable to those elicited by transfectants expressing the TNFR-corresponding TM TNFL (Medler et al., 2019). Obviously, the type/category of a TNFR strongly impacts the relevance of FcγR binding for agonistic antibody activity. The fact that category II TNFRs are superiorly activated by FcγR-bound antibodies can be straightforwardly explained in view of the two-step model of TNFR activation described above and the superior ability of TM versus soluble ligands to promote TNFR clustering: When a soluble TNFL molecule, which is able to recruit three TNF receptors, is not sufficient to promote secondary clustering of category II TNFRs to activate the classical NFκB pathway and cell death signaling, it is plausible that the two TNFR molecules that can be bound by an anti-TNFR IgG fail as well to constitute an active signaling complex. In a similar fashion to TM TNFLs, however, FcγR-bound anti-TNFR antibodies are presented in an “immobilized” plasma membrane-attached manner. Consequently, secondary clustering of complexes between FcγR-bound anti-TNFRs on FcγR^+^ anchor cells and TNFRs on TNFR^+^ target cells is envisaged in the cell-to-cell contact zone due to the high local concentrations of the molecules involved (Figure 6). FcγR-bound anti-TNFRs seem to mimic the superior ability of TM TNFRs to promote secondary clustering of liganded TNFR complexes. This concept suggests that the sole FcγR binding rather than the concrete epitope recognized by category II anti-TNFR antibodies is decisive for their agonistic activity. Indeed, on the example of antibody panels recognizing different epitopes on the category II TNFRs TNFR2, Fn14, CD40, and OX40, it has been found that FcγR binding and not the antibody idiotype is the decisive factor for agonistic activity (Salzmann et al., 2013; Trebing et al., 2014b; Dahan et al., 2016; Yu et al., 2018; Medler et al., 2019; Zhang P. et al., 2019). Systematic studies with anti-TNFR2 and anti-Fn14 antibodies furthermore suggested that any antibody–FcγR interaction, irrespective of the antibody isotype and FcγR type involved, results in significant activation of category II TNFRs, whereas without FcγR binding, none of the IgG isotypes display robust agonism (Medler et al., 2019). Thus, the isotype of an IgG antibody seems to be only of importance for the agonistic activity of anti-TNFR antibodies as long as it determines the ability to bind to FcγRs (Medler et al., 2019). It has been furthermore reported that wild-type and signaling defective FcγR mutants are equally effective in conferring agonism to anti-TNFR antibodies (Li and Ravetch, 2013). In sum, it can be asserted that it is the sheer cell surface attachment, thus the mimicry of the mode of presentation of the THD in TM TNFLs, that constitutes the agonism of FcγR-bound antibodies, while FcγR-specific activities are largely irrelevant. However, although anti-category II TNFR antibodies display *in vitro* a quite similar agonistic activity upon FcγR binding, this does not necessarily imply that there are no major differences in their *in vivo* activity. Thus, although anti-category II TNFR antibodies generally act as agonists upon FcγR binding, their concrete net effect *in vivo* can be different, especially under conditions where FcγR expression is limited and where the “free” non-FcγR-anchored antibody fraction therefore gains relevance. For example, the “free” antibody fraction may block TNFR binding by endogenous ligand molecules and/or compete with the agonistic FcγR-anchored antibody fraction for TNFR binding. Such factors might explain the finding that panels of antibodies against CD40 and OX40 have been found to be uniformly agonistic *in vitro* upon FcγR binding but show different agonistic potentials *in vivo* (Yu et al., 2018; Zhang P. et al., 2019).

If the plasma membrane-attached mode of presentation is indeed the crucial factor conferring a high agonistic potential to otherwise poorly active anti-TNFR antibodies, one has to expect that the agonism-releasing antibody–FcγR interaction can be replaced by other interactions which link the antibody to the plasma membrane. This seems to be indeed the case. Plasma membrane binding-dependent agonism has, for example, been demonstrated by different groups for anti-TRAILR2 antibody fusion proteins with the ability to anchor to the plasma membrane with the help of a second antibody domain recognizing the cell surface antigens FAP, MCSP, and FolR1 (Brunker et al., 2016; He et al., 2016; Shivange et al., 2018). Similarly, a 50- to >1,000-fold plasma membrane anchoring-dependent increase in their TNFR-stimulating potential has also been reported for various antibody fusion proteins targeting the category II TNFRs 4-1BB, CD27, CD40, CD95, Fn14, and TNFR2 (Medler et al., 2019; Nelke et al., 2020).

There are also a few examples of antibodies against the category II TNFRs CD40 and DR5 in the literature showing FcγR-independent agonism (Guo et al., 2005; Motoki et al., 2005; White et al., 2015; Yu et al., 2018). It has been claimed that the fully antibody-intrinsic agonism is due to the particular epitope recognized by these antibodies. However, it has not been addressed whether these antibodies are special by inducing TNFR clustering despite being only bivalent or whether these antibodies instruct the formation of fully signaling competent TNFR dimers that would be hard to reconcile with the knowledge on the mechanisms of TNFR activation. Worth mentioning, in further parallelism to poorly active trimeric complexes formed between soluble TNFL trimers and category II TNFRs, complexes of two TNFRs and an antibody gain high activity, when close proximity of the TNFR dimers is enforced by cross-linking or oligomerization of the antibody, e.g., with anti-IgG antibodies or protein G or protein A (Figure 6; Wajant, 2015).

Against the background of the great translational potential of agonists of category II TNFRs, it should be mentioned that the FcγR binding which is required for these antibodies to unfold their agonism comes along with effects limiting their applicability. First, possibly only a subfraction of TNFRs might be reached, activated *in vivo* due to poor availability of FcγR-expressing cells and/or low cellular FcγR expression levels. Second, antibody binding can trigger FcγR-mediated effects which counteract the therapeutic effects which are actually aspired with by the anti-TNFR antibody treatment. Third, considerable antibody doses are typically required to overcome competition with serum IgGs for FcγR binding. Last but not least and not intrinsically related to the need for FcγR binding, there can be dose-limiting side effects caused by the systemic activation of the targeted TNFR type [e.g., CD40: cytokine release/storm (Piechutta and Berghoff, 2019); TRAIL death receptors: hepatotoxicity (Papadopoulos et al., 2015; Zuch de Zafra et al., 2016; Nihira et al., 2019)]. Complications and limitations arising from the FcγR dependency of the agonism of anti-category II TNFR antibodies, however, might be straightforwardly circumvented by the use of antibody fusion proteins with an anchoring domain enabling FcγR-independent plasma membrane attachment as described above.

## Author Contributions

Both authors listed have made a substantial, direct and intellectual contribution to the work, and approved it for publication.

## Conflict of Interest

The authors declare that the research was conducted in the absence of any commercial or financial relationships that could be construed as a potential conflict of interest.
